# From Big Data to Precision Medicine

**DOI:** 10.3389/fmed.2019.00034

**Published:** 2019-03-01

**Authors:** Tim Hulsen, Saumya S. Jamuar, Alan R. Moody, Jason H. Karnes, Orsolya Varga, Stine Hedensted, Roberto Spreafico, David A. Hafler, Eoin F. McKinney

**Affiliations:** ^1^Department of Professional Health Solutions and Services, Philips Research, Eindhoven, Netherlands; ^2^Department of Paediatrics, KK Women's and Children's Hospital, and Paediatric Academic Clinical Programme, Duke-NUS Medical School, Singapore, Singapore; ^3^Department of Medical Imaging, University of Toronto, Toronto, ON, Canada; ^4^Pharmacy Practice and Science, College of Pharmacy, University of Arizona Health Sciences, Phoenix, AZ, United States; ^5^Department of Preventive Medicine, Faculty of Public Health, University of Debrecen, Debrecen, Hungary; ^6^Department of Molecular Medicine, Aarhus University Hospital, Aarhus, Denmark; ^7^Synthetic Genomics Inc., La Jolla, CA, United States; ^8^Departments of Neurology and Immunobiology, Yale School of Medicine, New Haven, CT, United States; ^9^Department of Medicine, University of Cambridge School of Clinical Medicine, Cambridge, United Kingdom

**Keywords:** big data, precision medicine, translational medicine, data science, big data analytics

## Abstract

For over a decade the term “Big data” has been used to describe the rapid increase in volume, variety and velocity of information available, not just in medical research but in almost every aspect of our lives. As scientists, we now have the capacity to rapidly generate, store and analyse data that, only a few years ago, would have taken many years to compile. However, “Big data” no longer means what it once did. The term has expanded and now refers not to just large data volume, but to our increasing ability to analyse and interpret those data. Tautologies such as “data analytics” and “data science” have emerged to describe approaches to the volume of available information as it grows ever larger. New methods dedicated to improving data collection, storage, cleaning, processing and interpretation continue to be developed, although not always by, or for, medical researchers. Exploiting new tools to extract meaning from large volume information has the potential to drive real change in clinical practice, from personalized therapy and intelligent drug design to population screening and electronic health record mining. As ever, where new technology promises “Big Advances,” significant challenges remain. Here we discuss both the opportunities and challenges posed to biomedical research by our increasing ability to tackle large datasets. Important challenges include the need for standardization of data content, format, and clinical definitions, a heightened need for collaborative networks with sharing of both data and expertise and, perhaps most importantly, a need to reconsider how and when analytic methodology is taught to medical researchers. We also set “Big data” analytics in context: recent advances may appear to promise a revolution, sweeping away conventional approaches to medical science. However, their real promise lies in their synergy with, not replacement of, classical hypothesis-driven methods. The generation of novel, data-driven hypotheses based on interpretable models will always require stringent validation and experimental testing. Thus, hypothesis-generating research founded on large datasets adds to, rather than replaces, traditional hypothesis driven science. Each can benefit from the other and it is through using both that we can improve clinical practice.

## Introduction

**Figure 1:**
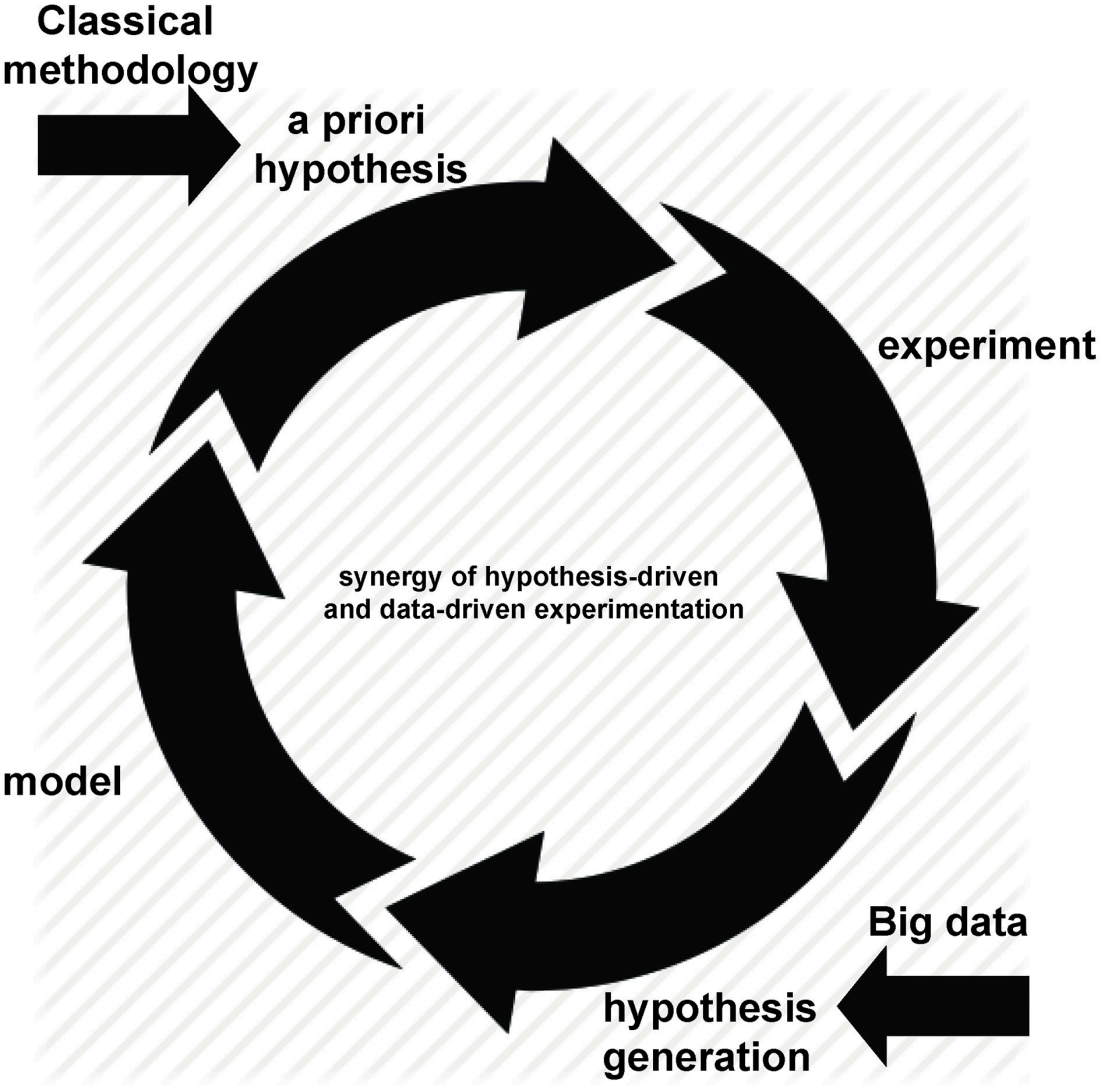
The synergistic cycle of hypothesis-driven and data-driven experimentation.

Advances in technology have created—and continue to create—an increasing ability to multiplex measurements on a single sample. This may result in hundreds, thousands or even millions of measurements being made concurrently, often combining technologies to give simultaneous measures of DNA, RNA, protein, function alongside clinical features including measures of disease activity, progression and related metadata. However, “Big data” is best considered not in terms of its size but of its purpose (somewhat ironically given the now ubiquitous use of the “Big” epithet; however we will retain the capital “B” to honor it). The defining characteristic of such experimental approaches is not the extended scale of measurement but the hypothesis-free approach to the underlying experimental design. Throughout this review we define “Big data” experiments as hypothesis generating rather than hypothesis driven studies. While they inevitably involve simultaneous measurement of many variables—and hence are typically “Bigger” than their counterparts driven by an *a priori* hypothesis—they do so in an attempt to describe and probe the unknown workings of complex systems: if we can measure it all, maybe we can understand it all. By definition, this approach is less dependent on prior knowledge and therefore has great potential to indicate hitherto unsuspected pathways relevant to disease. As is often the case with advances in technology, the rise of hypothesis-free methods was initially greeted with a polarized mixture of overblown enthusiasm and inappropriate nihilism: some believed that *a priori* hypotheses were no longer necessary (1), while others argued that new approaches were an irrelevant distraction from established methods (2). With the vantage point of history, it is clear that neither extreme was accurate. Hypothesis-generating approaches are not only synergistic with traditional methods, they are dependent upon them: after all, once generated, a hypothesis must be tested (Figure 1). In this way, Big data analyses can be used to ask novel questions, with conventional experimental techniques remaining just as relevant for testing them.

However, lost under a deluge of data, the goal of understanding may often seem just as distant as when we only had more limited numbers of measurements to contend with. If our goal is to understand the complexity of disease, we must be able to make sense of the complex volumes of data that can now be rapidly generated. Indeed, there are few systems more complex than those encountered in the field of biomedicine. The idea that human biology is composed of a complex network of interconnected systems is not new. The concept of interconnected “biological levels” was introduced in the 1940s (3) although a reductionist approach to biology can trace roots back as far as Descartes, with the analogy of deconstructing a clockwork mechanism prevalent from Newton (4) to Dawkins (5). Such ideas have informed the development of “systems biology,” in which we aim to arrive at mechanistic explanations for higher biological functions in terms of the “parts” of the biological machine (6).

The development of Big data approaches has greatly enhanced our ability to probe which “parts” of biology may be dysfunctional. The goal of precision medicine aims to take this approach one step further, by making that information of pragmatic value to the practicing clinician. Precision medicine can be succinctly defined as an approach to provide the right treatments to the right patients at the right time (7). However, for most clinical problems, precision strategies remain aspirational. The challenge of reducing biology to its component parts, then identifying which can and should be measured to choose an optimal intervention, the patient population that will benefit and when they will benefit most cannot be overstated. Yet the increasing use of hypothesis-free, Big data approaches promises to help us reach this aspirational goal.

In this review we summarize a number of the key challenges in using Big data analysis to facilitate precision medicine. Technical and methodological approaches have been systemically discussed elsewhere and we direct the reader to these excellent reviews (8–10). Here we identify key conceptual and infrastructural challenges and provide a perspective on how advances can be and are being used to arrive at precision medicine strategies with specific examples.

## Access and Technical Considerations for Harnessing Medical Big Data

The concept of Big data in medicine is not difficult to grasp: use large volumes of medical information to look for trends or associations that are not otherwise evident in smaller data sets. So why has Big data not been more widely leveraged? What is the difference between industries such as Google, Netflix and Amazon that have harnessed Big data to provide accurate and personalized real time information from on line searching and purchasing activities, and the health care system? Analysis of these successful industries reveals they have free and open access to data, which are provided willingly by the customer and delivered directly and centrally to the company. These deep data indicate personal likes and dislikes, enabling accurate predictions for future on-line interactions. Is it possible that large volume medical information from individual patient data could be used to identify novel risks or therapeutic options that can then be applied at the individual level to improve outcomes? Compared with industry, for the most part, the situation is different in healthcare. Medical records, representing deep private personal information, is carefully guarded and not openly available; data are usually siloed in clinic or hospital charts with no central sharing to allow the velocity or volume of data required to exploit Big data methods. Medical data is also complex and less “usable” compared with that being provided to large companies and therefore requires processing to provide a readily usable form. The technical infrastructure even to allow movement, manipulation and management of medical data is not readily available.

Broadly speaking, major barriers exist in the access to data, which are both philosophical and practical. To improve the translation of existing data into new healthcare solutions, a number of areas need to be addressed. These include, but are not limited to, the collection and standardization of heterogeneous datasets, the curation of the resultant clean data, prior informed consent for use of de-identified data, and the ability to provide these data back to the healthcare and research communities for further use.

### Industry vs. Medicine: Barriers and Opportunities

By understanding the similarities and the differences between clinical Big data and that used in other industries it is possible to better appreciate some opportunities that exist in the clinical field. It is also possible to understand why the uptake and translation of these techniques has not been a simple transfer from one domain to another. Industry uses data that can truly be defined as Big (exhibiting large volume, high velocity, and variety) but tends to be of low information density. These data are usually free, arising from an individual's incidental digital footprint in exchange for services. These data provide a surrogate marker of an activity that allows the prediction of patterns, trends, and outcomes. Fundamentally, data is acquired at the time services are accessed, with those data being either present or absent. Such data does exist in the clinical setting. Examples include physiological monitoring during an operation from multiple monitors providing high volume, high velocity and varied data that requires real time handling for the detection of data falling outside of a threshold that alerts the attending clinician. An example of lower volume data is the day to day accumulation of clinical tests that add to prior investigations providing updated diagnoses and medical management. Similarly, the handling of population based clinical data has the ability to predict trends in public health such as the timing of infectious disease epidemics. For these data the velocity provides “real time” prospective information and allows trend prediction. The output is referable to the source of the data, i.e., a patient in the operating room or a specific geographical population experiencing the winter flu season [Google Flu Trends (11)].

This real time information is primarily used to predict future trends (predictive modeling) without trying to provide any reasons for the findings. A more immediate target for Big data is the wealth of clinical data already housed in hospitals that help answer the question as to why particular events are occurring. These data have the potential, if they could be integrated and analyzed appropriately, to give insights into the causes of disease, allow their detection and diagnosis, guide therapy, and management, plus the development of future drugs and interventions. To assimilate this data will require massive computing far beyond an individual's capability thus fulfilling the definition of Big data. The data will largely be derived from and specific to populations and then applied to individuals (e.g., patient groups with different disease types or processes provide new insights for the benefit of individuals), and will be retrospectively collected rather than prospectively acquired. Finally, while non-medical Big data has largely been incidental, at no charge and with low information density, the Big data of the clinical world will be acquired intentionally, costly (to someone) with high information density. This is therefore more akin to business intelligence which requires Big data techniques to derive measurements, and to detect trends (not just predict them), which are otherwise not visible or manageable by human inspection alone.

The clinical domain has a number of similarities with the business intelligence model of Big data which potentially provides an approach to clinical data handling already tested in the business world.

Within the electronic health record, as in business, data are both structured and non-structured and technologies to deal with both will be required to allow easy interpretation. In business, this allows the identification of new opportunities and development of new strategies which can be translated clinically as new understanding of disease and development of new treatments. Big data provides the ability to combine data from numerous sources both internal and external to business; similarly, multiple data sources (clinical, laboratory tests, imaging, genetics, etc.) may be combined in the clinical domain and provide “intelligence” not derived from any single data source, invisible to routine observation. A central data warehouse provides a site for integrating this varied data allowing curation, combination and analysis. Currently such centralized repositories do not commonly exist in clinical information technology infrastructure within hospitals. These data repositories have been designed and built in the pre-Big data era being standalone and siloed, with no intention of allowing the data to be combined and then analyzed in conjunction with various data sets. There is a need for newly installed information technology systems within clinical domains to ensure there is a means to share data between systems.

### Philosophy of Data Ownership

Patient data of any sort, because it is held within medical institutions, appears to belong to that institution. However, these institutions merely act as the custodians of this data—the data is the property of the patient and the access and use of that data outside of the clinical realm requires patient consent. This immediately puts a brake on the rapid exploitation of the large volume of data already held in clinical records. While retrospective hypothesis driven research can be undertaken on specific, anonymized data as with any research, once the study has ended the data should be destroyed. For Big data techniques using thousands to millions of data points, which may have required considerable processing, the prospect of losing such valuable data at the end of the project is counter-intuitive for the advancement of medical knowledge. Prospective consent of the patient to store and use their data is therefore a more powerful model and allows the accumulation of large data sets then allowing the application of hypothesis driven research questions to those data. While not using the vast wealth of retrospective data feels wasteful, the rate (velocity) at which new data are accrued in the medical setting is sufficiently rapid that the resultant consented data is far more valuable. This added step of acquiring consent from patients likely requires on site manpower to interact with patients. Alternatively, options such as patients providing blanket consent for use of their data may be an option but will need fully informed consent. This dilemma has been brought to the fore by the EU General Data Protection Regulation (GDPR) which entered into force in 2018, initiating an international debate on Big data sharing in health (12).

### Regulations Regarding Data Sharing

On April 27, 2016, the European Union approved a new set of regulations around privacy: the General Data Protection Regulation (GDPR) (13), which is in effect since May 25, 2018. The GDPR applies if the data controller (the organization that collects data), data processor (the organization that processes data on behalf of the data controller) or data subject is based in the EU. For science, this means that all studies performed by European institutes/companies and/or on European citizens, will be subject to the GDPR, with the exception of data that is fully anonymized (14). The GDPR sets out seven key principles: lawfulness, fairness and transparency; purpose limitation; data minimization; accuracy; storage limitation; integrity and confidentiality (security); and accountability. The GDPR puts some constraints on data sharing, e.g., if a data controller wants to share data with another data controller, he/she needs to have an appropriate contract in place, particularly if that other data controller is located outside the EU (15). If a data controller wants to share data with a third party, and that third party is a processor, then a Data Processor Agreement (DPA) needs to be made. Apart from this DPA, the informed consent that the patient signs before participating in a study, needs to state clearly for what purposes their data will be used (16). Penalties for non-compliance can be significant, GDPR fines are up to €20 million or 4% of annual turnover. Considering the fact that health data are “sensitive,” potential discrimination has been addressed in legislation and a more proportionate approach is applied to balance privacy rights against the potential benefits of data sharing in science (12). In fact, processing of “data concerning health,” “genetic data,” and “biometric data” is prohibited unless one of several conditions applies: data subject gives “explicit consent,” processing is necessary for the purposes of provision of services or management of health system (etc.), or processing is necessary for reasons of public interest in the area of public health.

The question of “explicit consent” of patients for their healthcare data to be used for research purposes provoked intense debate already during negotiations for GDPR, but finally these research groups lost the argument in the European political arena to advocacy groups of greater privacy. Research groups lobbied that restricting access to billions of terabytes of data would hold back research e.g., into cancer in Europe. The fear as concluded by Professor Subhajit Basu, from Leeds University, is that “GDPR will make healthcare innovation more laborious in Europe as lots of people won't give their consent. It will also add another layer of staff to take consent, making it more expensive. We already have stricter data protection laws than the US and China, who are moving ahead in producing innovative healthcare technology.” (17)

Within the GDPR, the data subject also has the “right to be forgotten”: he/she can withdraw the consent, after which the data controller needs to remove all his/her personal data (18). Because of all issues around data sharing, scientists might consider (whenever possible) to share only aggregated data which cannot be traced back to individual data subjects, or to raise the abstraction level, sharing insights instead of data (19).

While the implementation of GDPR has brought this issue into sharp focus, it has not resolved a fundamental dilemma. As clinicians and scientists, we face an increasingly urgent need to balance the opportunity Big data provides for improving healthcare, against the right of individuals to control their own data. It is our responsibility to only use data with appropriate consent, but it is also our responsibility to maximize our ability to improve health. Balancing these two will remain an increasing challenge for all precision medicine strategies.

### Sharing of Data, Experience and Training—FAIR Principles

The sharing of data only makes sense when these data are structured properly [preferably using an ontology such as BFO, OBO, or RO (20)], contains detailed descriptions about what each field means (metadata) and can be combined with other data types in a reliable manner. These tasks are usually performed by a data manager or data steward (21), a function that has been gaining importance over the past years, due to the rise of “Big data.” Until recently, data managers and data stewards had to do their job without having a clear set of rules to guide them. In 2016 however, the FAIR Guiding Principles for scientific data management and stewardship (22) were published. FAIR stands for the four foundational principles—Findability, Accessibility, Interoperability, and Reusability—that serve to guide data producers and publishers as they navigate around the obstacles around data management and stewardship. The publication offers guidelines around these four principles (Table 1).

**Table 1:** FAIR principles for data management and stewardship.

Findability: (meta)data are assigned a globally unique and persistent identifier; data are described with rich metadata; metadata clearly and explicitly include the identifier of the data it describes; (meta)data are registered or indexed in a searchable resource Accessibility: (meta)data are retrievable by their identifier using a standardized communications protocol; this protocol is open, free, and universally implementable, and allows for an authentication and authorization procedure, where necessary; metadata are accessible, even when the data are no longer available Interoperability: (meta)data use a formal, accessible, shared, and broadly applicable language for knowledge representation; they use vocabularies that follow FAIR principles; they include qualified references to other (meta)data Reusability: (meta)data are richly described with a plurality of accurate and relevant attributes; they are released with a clear and accessible data usage license; they are associated with detailed provenance; they meet domain-relevant community standards

### Physical Infrastructure

On first impression, healthcare institutions are well equipped with information technology. However, this has been designed to support the clinical environment and billing, but not the research environment of Big data. Exploitation of this new research environment will require a unique environment to store, handle, combine, curate and analyse large volumes of data. Clinical systems are built to isolate different data sets such as imaging, pathology and laboratory tests, whereas the Big data domain requires the integration of data. The EHR may provide some of this cross referencing of unstructured data but does not give the opportunity for deriving more complex data from datasets such as imaging and pathology which gives the opportunity for further analysis beyond the written report. To do this, as mentioned above, a data warehouse provides a “third space” for housing diverse data that normally resides elsewhere. This allows the handling of multiple individuals at the same time, grouped by some common feature such as disease type or imaging appearance, which is the opposite of a clinical system which usually is interested in varied data from one patient at a time.

A data warehouse allows secondary handling to generate cleaner, more information-rich data as seen when applying annotations and segmentation in pathological and radiological images. In order to achieve this, the data warehouse needs to provide the interface with multiple software applications. Within the warehouse, the researcher can gather varied, high volume data that can then undergo various pre-processing techniques in readiness for the application of Big data techniques including artificial intelligence and machine learning. The latter needs specialized high-powered computing to achieve rapid processing. Graphic processing units (GPUs) allow the handling of very large batches of data, and undertake repetitive operations that can accelerate processing by up to a hundred times compared to standard central processing units (CPU's). As previously stated, current data handling systems are not yet equipped with these processors requiring upgrading of hardware infrastructure to translate these new techniques into the clinical domain. The connection of these supercomputing stacks to the data can potentially be achieved via the central data warehouse containing the pre-processed data drawn from many sources.

### Clinical Translation

A significant barrier to the application of new Big data techniques into clinical practice is the positioning of these techniques in the current clinical work environment. New and disruptive technologies provided by Big data analytics are likely to be just that… disruptive. Current clinical practice will need to undergo change to incorporate these new data driven techniques. There may need to be sufficient periods of testing of new techniques, especially those which in some way replace a human action and speed the clinical process. Those that aid this process by prioritizing worklists or flagging urgent findings are likely to diffuse rapidly into day to day usage. Similarly, techniques not previously possible because of the sheer size of data being handled are likely to gain early adoption. A major player in achieving this process will be industry which will enable the incorporation of hardware and software to support Big data handling in the current workflow. If access to data and its analysis is difficult and requires interruption of the normal clinical process, uptake will be slow or non-existent. A push button application on a computer screen however, as on an x-ray image viewer that seamlessly activates the program in the background is far more likely to be adopted. Greater success will be achieved with the automatic running of programs triggered by image content and specific imaging examinations. As previously mentioned, these programs could potentially provide identification of suspicious regions in complex images requiring further scrutiny or undertake quantitative measurements in the background which are then presented through visualization techniques in conjunction with the qualitative structured report. Furthermore, quantitative data can then be compared with that from large populations to define its position in the normal range and, from this and other clinical data, provide predictive data regarding drug response, progression and outcome. Part of the attraction for industry in this rapidly expanding arena will obviously be the generation of Intellectual Property (IP). Development of new techniques useful to clinical departments will require close collaboration between industry and clinical researchers to ensure developments are relevant and rigorously tested in real life scenarios.

IP protection—provided mainly via patents—is the pillar of national research policies and essential to effectively translate innovation by commercialization. In the absence of such protection, companies are unlikely to invest in the development of diagnostic tests or treatments (23). However, the operation of the IP system is being fundamentally changed by Big data based technical solutions. Due to free and open source software tools for patent analytics (24) and technical advances in patent searches such as visualization techniques it is easy to gain access to high quality inventions and intellectual property being developed and to understand the findings. Today, unlike a traditional state-of-the-art search which provides relevant information in text format, patent landscape analysis provides graphics and charts that demonstrate patenting trends, leading patent assignees, collaboration partners, white space analysis, technology evaluations (25). By using network based and Big data analysis, important patent information including owner, inventor, attorney, patent examiner or technology can be determined instantly. Presently, patent portfolios are being unlocked and democratized due to free access to patent analysis. In the near future, automated patent landscaping may generate high-quality patent landscapes with minimal effort with the help of heuristics based on patent metadata and machine learning thereby increasing the access to conducting patent landscape analysis (26). Although such changes within the operation of the IP system gives new possibilities to researchers, it is hard to forecast the long-term effect, whether and how incentives in health research will be shifted (encouraging/discouraging innovation).

### The Bigger the Better? Challenges in Translating From Big Data

Just as the volume of data that can be generated has increased exponentially, so the complexity of those data have increased. It is no longer enough to sequence all variants in a human genome, now we can relate them to transcript levels, protein levels, metabolites or functional and phenotypic traits also. Moreover, it has become clear that reconstruction of single cell data may provide significantly more insight into biological processes as compared to bulk analysis of mixed populations of cells (27). It is now possible to measure concurrent transcriptomes and genetics [so-called G&T seq (28)] or epigenetic modifications (29) on a single cell. So, as the volume of data increases, so does its complexity. Integrating varied Big data from a set of samples, or from a partially overlapping set of samples, has become a new frontier of method development. It is not the goal of this review to provide a comprehensive review of such methods [which have been comprehensively and accessibly reviewed elsewhere (8)] but instead to highlight core challenges for generating, integrating, and analysing data such that it can prove useful for precision medicine.

### Forged in the Fire of Validation: The Requirement for Replication

While new technologies have greatly increased our ability to generate data, old problems persist and are arguably accentuated by hypothesis-generating methods. A fundamental scientific tenet holds that, for a result to be robust, it must be reproducible. However, it has been reported that results from a concerning proportion of even the highest ranking scientific articles may not be reproduced [~11% only were (30)]. Granted this was a restricted set of highly novel preclinical findings and the experimental methods being used were in some cases advanced enough to require a not always accessible mastery/sophistication for proper execution. Still, the likelihood that such independent validation will even be attempted, let alone achieved, inevitably falls as the time, energy and cost of generating data increases. Big data is often expensive data but we should not allow a shift toward hypothesis-free experimentation to erode confidence in the conclusions being made. The best—arguably the only—way to improve confidence in the findings from Big data is to work to facilitate transparent validation of findings.

**Figure 2:**
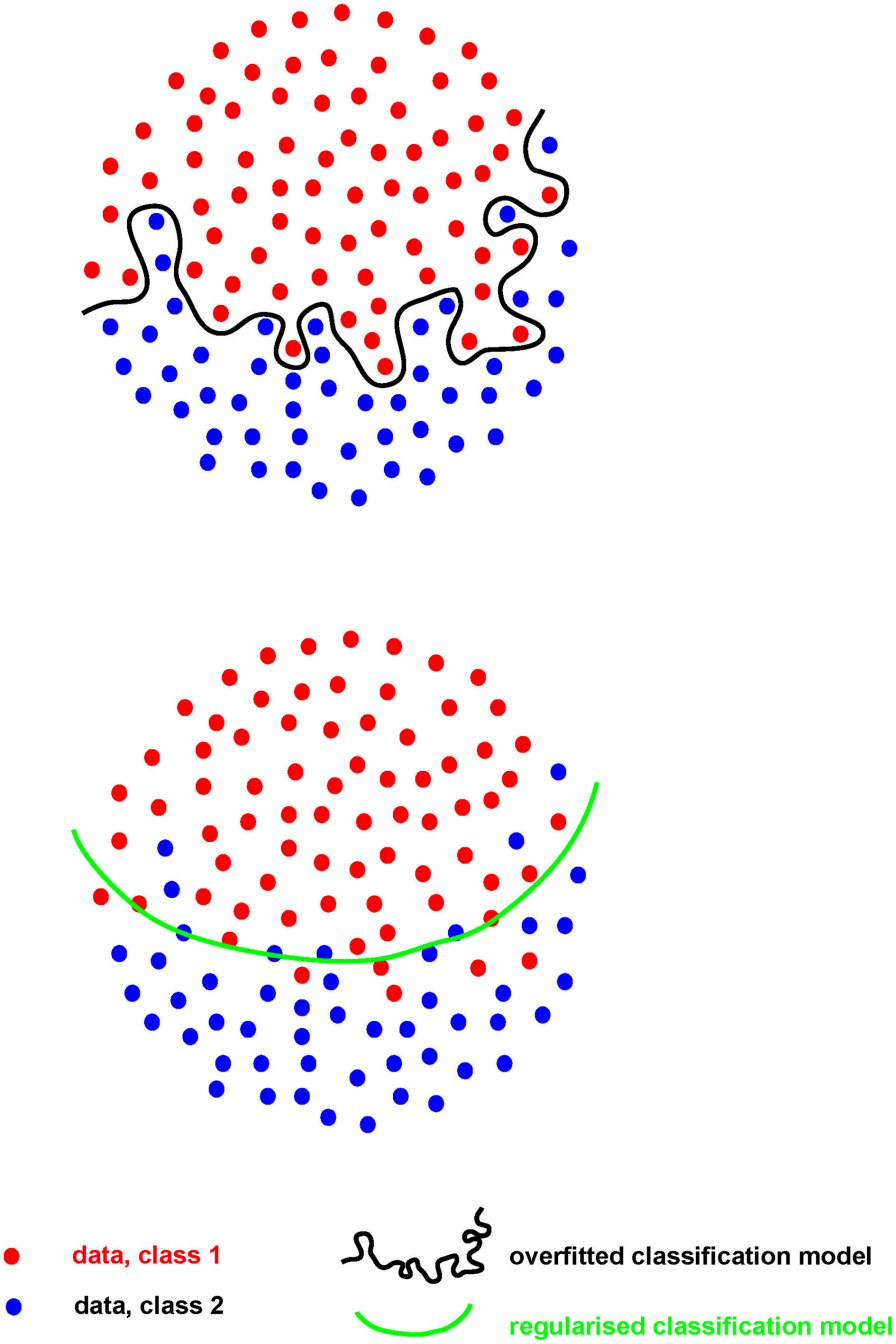
The concept of overfitting and model regularization.

Where the number of measures (p) greatly outstrips the number of samples they are made on (n), the risk of “overfitting” becomes of paramount importance. Such “p >> n” problems are common in hypothesis-generating research. When an analytical model is developed, or “fitted,” on a set of Big data (the “training” set), the risk is that the model will perform extremely well on that particular dataset, finding the exact features required to do so from the extensive range available. However, for that model to perform even acceptably on a new dataset (the “test” set), it cannot fit the training set *too* well (i.e., be “over-fitted,” Figure 2). A model must be found that both reflects the data and is likely to generalize to new samples, without compromising too much the performance on the training set. This problem, called model “regularization,” is common to many machine-learning approaches (31) and is of increasing importance as data volume and complexity increases.

**Figure 3:**
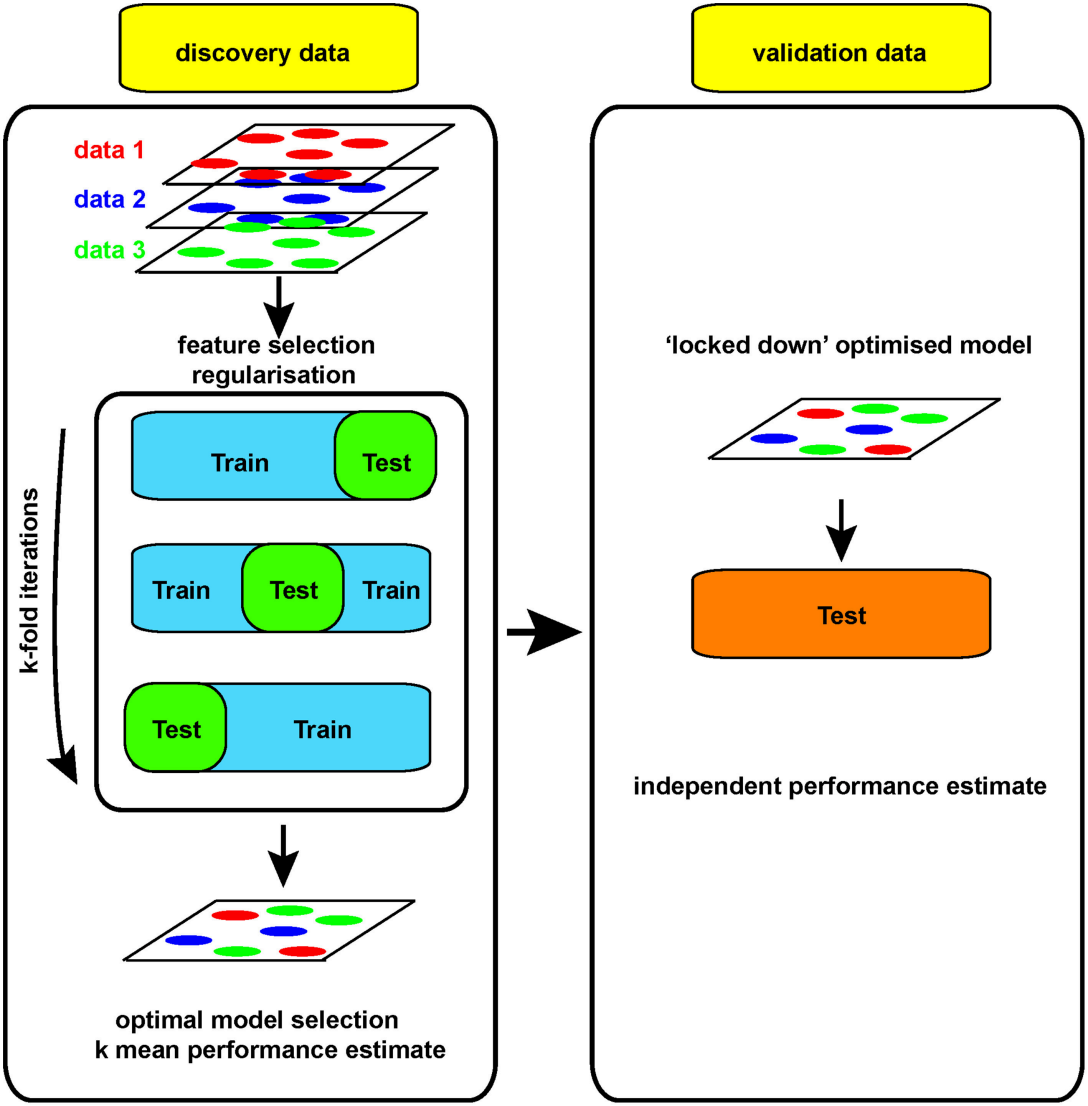
Model performance evaluation.

For example, if a large dataset (perhaps 100 k gene expression measures on 100 patients/controls by RNA sequencing) is analyzed, a clear model may be found using 20 genes whose expression allows clean separation of those with/without disease. This may appear to be a useful advance, allowing clean diagnosis if the relevant genes are measured. While the result is encouraging, it is impossible to tell at this stage whether the discriminating model will prove useful or not: its performance can only be assessed on samples that were not used to generate the model in the first place. Otherwise, it looks (and is) too good to be true. Thus, replication in a new, independent data set is absolutely required in order to obtain a robust estimate of that models performance (Figure 3).

While this example is overly simplistic, overfitting remains an insidious obstacle to the translation of robust, reproducible hypotheses from biomedical Big data. Overfitting occurs easily and is all the more dangerous because it tells us what we want to hear, suggesting we have an interesting result, a strong association. Risk of overfitting is also likely to increase as the complexity of data available increases, i.e., as *p* increases. To counter that, increasing the number of independent samples (*n*) becomes fundamental. As surprising at it may seem, in the early days of RNA-seq, experiments were performed with no replicates, leading to prominent genomic statisticians to remind the community that next-generation sequencing platforms are simply high-throughput measurement platforms still requiring biological replication (32). As we accumulate genomics data of several kinds across a spectrum of diseases, estimating effect sizes becomes more accurate and more accessible. In turn, realistic effect sizes enable *a priori* power calculations and/or simulations, which help reveal whether a study is sufficiently sensitive, or rather doomed by either false negatives or spurious correlations.

Yet generating sufficient samples and funds to process independent cohorts can be challenging at the level of an individual academic lab. That is why performing science at the scale of networks of labs or even larger international consortia is essential to generate reliable, robust and truly *big* (as in *big n*, not *big p*) biomedical/genomic datasets. Funding agencies have widely embraced this concept with collaborative funding models such as the U and P grant series, and large-scale initiatives such as ENCODE, TCGA, BRAIN from the NIH or Blueprint in the EU, to mention just a few. Training datasets need to be large enough to allow discovery, with comparable test datasets available for independent validation. Leave-one-out cross-validation approaches (LOOCV, where one sample is re-iteratively withheld from the training set during model development to allow an independent estimate of performance) are useful and can be built into regularization strategies (31) (Figure 3). However, changes in the way the biomedical community works, with increasing collaboration and communication is also facilitating validation of models built on Big data.

### Community Service: The Bigger the Better?

The development of international consortia and networks facilitating sample collection and distribution facilitate access to precious biomaterials from patients. Such biorepositories can provide access to carefully annotated samples, often including a wide range of assay materials and with detailed metadata. This can rapidly expand the pool of samples available for the generation of large datasets. Examples include the TEDDY and TrialNet consortia in diabetes (33, 34), the UK Biobank (35) and the Immune Tolerance Network (ITN), in which samples from completed clinical trials can be accessed by collaborators in conjunction with detailed clinical metadata through the TrialShare platform (36). This allows clinical trials to create value far beyond answering a focused primary or restricted secondary endpoints. This model is further extended in attempts to build similar biorepositories from pharmaceutical industry trials in which the placebo arms can be compiled into a single meta-study for new discovery work (37). A positive side-effect of large international consortia is that the complexity of coordination requires developing well-defined standard operating procedures (SOPs) for both experimental and analytical procedures. Because consortia group together so many experts in a particular field, the resulting (public) SOPs often become the standard *de facto* resource for the task at hand. For example, ChIP-seq protocols from the ENCODE initiative (http://www.encodeproject.org/data-standards), as well as its bioinformatics pipelines (http://www.encodeproject.org/pipelines), are often referred to by a constellation of published studies having no formal connection with the primary initiative.

It is not just biosamples that are increasingly being shared, but also data. In addition to the example networks above, the Cancer Genome Atlas has generated—and made publicly available—integrated molecular data on over 10,000 samples from 33 different tumor types. This huge, collaborative project not only references genetic, epigenetic, transcriptomic, histologic, proteomic, and detailed clinical data but does so in the context of an accessible data portal facilitating access and use (38). Most importantly, there is clear evidence that this approach can work, with cancer-associated mutation identification driving target identification and precision medicine trials (39).

Forming a community of researchers can do more than simply collect samples to be used in data generation. Harnessing the analytical experience of a community of researchers is the explicit goal of crowdsourcing approaches such as that used by the DREAM challenges [Dialogue for Research Engineering Assessments and Methods (40)]. This alternative model of data sharing and analysis effectively reverses the conventional flow of data between repositories and analysts. Community sourced, registered users can access data and effectively compete against each other to train optimal models. Importantly, the ultimate “winner” is determined by validation on independent “containerized” datasets, controlling the risk of overfitting during development. This innovative approach has facilitated not only broader access to key datasets but has also engaged the analytic community broadly with notable successes (41). Indeed, following centralized cloud-based processing of several challenges, consistent themes have emerged from successful models. Core principles of successful models have been their simplicity and inclusion of approaches either integrating different models or prior knowledge of the relevant field (8). In particular, this last observation has important ramifications for the integration of biomedical and analytical training (see below).

Arguably the most remarkable success in the field of Big data receives almost no attention. Despite significant potential for the creation of protectable value, software developers have almost universally made source code freely available through open-source tools that are frequently adapted and improved by the scientific community (42). Encouraged by organizations such as the Open Bioinformatics Foundation and the International Society for Computational Biology, this quiet revolution has undoubtedly had a huge impact on the rate of progress and our ability to harness the potential of Big data and continues to do so.

### Setting Standards

In order for robust validation to work, it is necessary to ensure that measurements made in a training cohort are comparable to those made in a test set. This sounds straightforward, but isn't. Robust analysis can fail to validate just as easily as overfitted analysis, particularly where patients may come from different healthcare systems, samples may be collected in different ways and data may be generated using different protocols.

While there has been remarkable progress in detection of disease using a single blood sample (such as genome sequencing in affected individuals, circulating cell free tumor DNA in oncology, non-invasive prenatal testing in pregnancy, among others), it is no longer enough to provide all the information about an individual with respect to one's health. There is an increased understanding of the need for repeat sampling to gather longitudinal data, to measure changes over time with or without a significant exposure (43, 44). More importantly, the ability to interrogate single cells has spurred a need to identify and isolate the tissue of interest and select appropriate samples from within that tissue (45). Tissue samples include blood, saliva, buccal swabs, organ-specific such as in tumors, as well as stool samples for the newly emerging field of microbiome.

Over the past two decades, methods have been established to ensure standardization of extracted genomic material such as DNA from blood and other fresh tissues, including automation (46). However, other samples such as DNA from paraffin embedded tissue, RNA, protein are more sensitive to type of tissue and tissue handling, and may not be robust enough for replication studies. For Big data science to work, one of the key ingredients is robust and reproducible input data. In this regard, there have been recent advancements in attempts to standardize the way these samples are collected for generating “omics” data (47–49). Basic experimental methodologies involved in sample collection or generation are crucial for the quality of genomics datasets, yet, in practice, they are often neglected. Twenty-First century omics-generating platforms are often perceived to be so advanced and complex, particularly to the novice, that should draw most of the planning effort, leaving details on trivial Twentieth-century steps (cell culture, cell freezing, nucleic acid isolation) comparatively overlooked. If anything, while experiments performed on poorly generated material would yield only a handful of flawed data points in the pre-genomics era, they would bias thousands of data points at once in the big data era. When thinking at the reasons underlying failed or low-quality omics experiments in daily practice, it is easy to realize that trivialities and logistics played a major role, while sequencing and proteomics platforms are seldom the culprit.

Similar considerations apply when choosing the starting biologic material for a big data experiments. Because of its accessibility, blood is still the most widely available tissue in human research and is often well suited for investigating immune-related diseases. However, peripheral blood mononuclear cells (PBMCs) are a complex mixture of immune cell types. Immune profiling using omics technologies is overwhelmingly performed on total PBMCs, as opposed to more uniform cell subsets. While cutting-edge single-cell platforms can deal with complex mixtures of cell types, the more widespread bulk platforms, such as Illumina's sequencers, can only measure averages from a cell population. Unfortunately, such averages are a function of both differential regulation of mechanisms of interest (such as gene expression) and the often-uninteresting differential prevalence of each cell type—this latter effectively qualifying as unwanted noise, from an experimentalist's standpoint. This issue is often either unappreciated or disregarded as solvable by deconvolution algorithms, that is software that attempts to recover the unmeasured signal from contributing subsets to the measured mean. However, deconvolution software often deals with just the main white blood cell subsets, leading to coarse resolution; is usually trained on healthy controls, limiting its usefulness in diseases substantially altering molecular fingerprints, such as cancer; and its accuracy is severely limited (an extreme example being CD4+ and CD8+ T cells, lymphocytes with a clear immunological distinction, but sharing so much of the epigenetics and transcriptional landscape to be very hard to deconvolve separately) (50, 51).

Lastly, standardization of samples also allows for data generated from one individual/ cohort to be used in other related studies and obviates the need to generate the same data over and over again. While this has to be within the remit of ethics and data sharing regulations for each institution/ country, it allows for better use of limited resources (such as clinical material) and funds.

### Data Comparability

The past decade has seen remarkable progress in development of standard genomic data formats including FASTQ, BAM/CRAM, and VCF files (52). However, such standardization is incomplete and may lead to incompatibility between inputs and outputs of different bioinformatics tools, or, worse, inaccurate results. An example is the quality encoding format of FASTQ files, which is not captured by the file itself, and must be either inferred or transmitted as accompanying metadata with the file itself. Still, even an imperfect standardization has allowed for sharing of genomic data across institutions into either aggregated databases such as ExAC, GNOMAD (53) or as federated database such as Beacon Network (54). These databases allow for understanding of genetic variations that are common across different ethnic groups but also identifies variants that are unique within a specific ethnic group (53). However, despite these successes with upstream genomic data formats, key challenges remain regarding further downstream data formats. This often leads to non-uniform analysis, and indeed, re-analysis of the same data using different pipelines yields different results (55, 56).

Similar efforts have been developed in the field of proteomics (57) and microbiomics (49). In view of the increasing recognition of a need for such standards. The American College of Medical Genetics released guidelines to aid interpretation of genomic variants (58), the ClinGen workgroup has released a framework to establish gene-disease relationship (59) and Global Alliance for Genomics and Health (GA4GH), in collaboration with National Institute of Health (NIH), have developed genomic data toolkit which includes standards for storage and retrieval of genomic data (54).

### Clinical and Phenotypic Definitions

One of the largest challenges with harmonizing Big data is definition of cases (disease) and controls (health) (60). Stating the obvious, no one is healthy for ever. Using strict definitions based on consensus statements allow comparability of diseases across different populations. There have been several initiatives to standardize phenotypic terminology including Human Phenotype Ontology (HPO), Monarch Initiative, among others (61, 62). In addition, standard diagnostic codes such as SNOMED CT, ICD-10, etc. provide for computer processable codes which standardize medical terms and diagnosis, and lead to consistent information exchange across different systems (63). As we move toward use of machine learning and artificial intelligence, the use of controlled vocabularies is critical. Even more important is the need for robust definitions of the clinical phenotypes and diagnosis that accompany these samples so as to ensure accurate comparison between cases and controls. The often heard phrase “Garbage in, Garbage out” is ever more relevant in the days of Big data science (64). Establishing clear principles on data access and sharing is a key step in establishing and maintaining community-wide access to the kind of collaborative sample sharing required to facilitate both discovery and validation.

### Opportunities for Clinical Big Data: Leveraging the Electronic Health Record

The EHR is an intrinsically large resource as the majority of patients in the developed world are treated in this context. There is a staggering amount of information collected longitudinally on each individual, including laboratory test results, diagnoses, free text, and imaging. This existing wealth of information is available at virtually zero cost, collected systematically for decades. Whereas the EHR has classically been used in clinical care, billing, and auditing, it is increasingly used to generate evidence on a large scale (65). Population-based studies tend to be disease-specific, but the EHR is largely disease agnostic. Thus the EHR provides opportunities to study virtually any disease as well as pleiotropic influences of risk factors such as genetic variation. Since the EHR was not originally designed for evidence generation, leveraging these data is fraught with challenges related to the collection, standardization, and curation of data. While opportunities exist to study a spectrum of phenotypes, data contained in the EHR is generally not as rigorous or complete as that collected in a cohort-based study. Nevertheless, these EHRs provide potential solutions to problems involving Big data, including the reliability and standardization of data and the accuracy of EHR phenotyping. As discussed below, there are multiple examples of how these challenges are being met by researchers and clinicians across the globe.

Among the formidable challenges related to leveraging the resources of the EHR is assurance of data quality. Missing data abounds in these records and the study of many conditions relies on mining narrative text with natural language processing rather than more objective testing such as laboratory measures and genomic sequencing. Misclassification is often encountered within the electronic health record such as with International Classification of Diseases-10th Revision (ICD-10) codes. EHR data would also be improved by recording of lifestyle choices such as diet and exercise, family history and relationships between individuals, race and ethnicity, adherence to prescribed drugs, allergies, and data from wearable technologies. Standardization of data is also an issue. The EHR includes structured data such as Systematized Nomenclature of Medicine-Clinical Terms (SNOMED CT) terms and ICD-10 codes as well as unstructured data such as medical history, discharge summaries, and imaging reports in unformatted free text (65). Standardization across multiple countries and EHR software tools provides a vast opportunity for scalability. As the technical issues with EHR data are addressed, legal and ethical frameworks will be necessary to build and maintain public trust and ensure equitable data access.

Despite the many challenges that have yet to be addressed, the EHR provides a wide variety of opportunities for improving human health. The wealth of existing data that the EHR provides enables richer profiles for health and disease that can be studied on a population level. There is much effort in standardization of EHR phenotyping, which has the potential to create sub-categories of disease and eventually re-taxonomise diseases. (66, 67) The EHR affords an opportunity for efficient and cost effective implementation, allowing efficient return of data to patient and provider. The integration of pharmacogenomics testing into patient care is an example of the translational power of the EHR (68, 69).

EHR data is increasingly being coupled to biorepositories, creating opportunities to leverage “-omic” data in combination with EHR phenotyping. Noteworthy examples include the UK biobank and eMERGE Network (35, 70). With these new resources, new and innovative tools are being developed, such as the phenome-wide association study (PheWAS) (71, 72). Using the PheWAS approach, genomic variation form biorepository is systematically tested for association across phenotypes in the EHR. The PheWAS approach presents a useful approach to assess pleiotropy of genomic variants, allows the study of human knockouts, and is a new approach to drug discovery and drug repurposing. The coupling of EHR and -omic data also enables translation of discovery back to the clinic. Such biorepositories can mine genomic variation to confirm disease diagnoses or re-diagnose/re-classify patients in a clinical setting. In a recent study, combinations of rare genetic variants were used to identify subsets of patients with distinct genetic causes for common diseases that suffered severe outcomes such as organ transplants (73). While illustrating the power of these biorepositories, it is worth noting that these results were not returnable to patients due to restrictions around the ethical approval of the biorepository.

### Artificial Intelligence and Clinical Imaging

While health improvements brought about by the application of Big data techniques are still, largely, yet to translate into clinical practice, the possible benefits of doing so can be seen in those clinical areas already with large, easily available and usable data sets. One such area is in clinical imaging where data is invariably digitized and housed in dedicated picture archiving systems. In addition, this imaging data is connected with clinical data in the form of image reports, the electronic health record and also carries its own metadata. Because of the ease of handling of this data, it has been possible to show, at least experimentally, that artificial intelligence through machine learning techniques, can exploit Big data to provide clinical benefit. The need for these high-powered computing techniques in part reflects the need to extract hidden information from images which is not readily available from the original datasets. This is in contrast to simple parametric data within the clinical record including physiological readings such as pulse rate or blood pressure, or results from blood tests. The need for similar data processing is also seen in digitized pathology image specimens.

Big data can provide annotated data sets to be used to train artificial intelligence algorithms to recognize clinically relevant conditions or features. In order for the algorithm to learn the relevant features, which are not pre-programmed, significant numbers of cases with the feature or condition under scrutiny are required. Subsequently, similar, but different large volumes of cases are used to test the algorithm against gold standard annotations. Once trained to an acceptable level these techniques have the opportunity to provide pre-screening of images to look for cases with high likelihood of disease allowing prioritization of formal reading. Screening tests such as breast mammography could undergo pre-reading by artificial intelligence/machine learning to identify the few positive cases among the many normal studies allowing rapid identification and turnaround. Pre-screening of complex high acuity cases as seen in the trauma setting also allow a focused approach to identify and review areas of concern as a priority. Quantification of structures within an image such as tumor volume, monitoring growth or response to therapy, or cardiac ejection volume, to manage drug therapy of heart failure or following heart attack, can be incorporated into artificial intelligence/machine learning algorithms so they are undertaken automatically rather than requiring painstaking manual segmentation of structures.

As artificial intelligence/machine learning continues to improve it has the ability to recognize image features without any pre-training through the application of neural networks which can assimilate different sets of clinical data. The resultant algorithms can then be applied to similar, new clinical information to predict individual patient responses based on large prior patient cohorts. Alternatively, similar techniques can be applied to images to identify sub populations that are otherwise too complex to recognize. Furthermore, artificial intelligence/machine learning may find a role in hypothesis generation by identifying unrecognized, unique image features or combination of features that relate to disease progression or outcome. For instance, a subset of patients with memory loss that potentially progress to dementia may have features detectable prior to symptom development. This approach allows large volume population interrogation with prospective clinical follow up and identification of the most clinically relevant image fingerprints, rather than analysis of small volume retrospective data in patients who have already developed symptomatic degenerative brain disease.

Despite the vast wealth of data contained in the clinical information technology systems within hospitals, extraction of usable data from the clinical domain is not a trivial task. This is for a number of diverse reasons including: philosophy of data handling; physical data handling infrastructure; the data format; and translation of new advances into the clinical domain. These problems must be addressed prior to successful application of these new methodologies.

### New Data, New Methods, New Training

It is clear that sample and phenotypic standardization provide clear opportunities to add value and robust validation through collaboration. However, increasing availability of data has been matched by a shortage of those with the skills to analyse and interpret those data. Data volume has increased faster than predicted and, although the current shortage of bioinformaticians was foreseen (74), corrective measures are still required to encourage skilled analysts to work on biomedical problems. Including prior knowledge of relevant domains demonstrably improves the performance of models built on Big data (8, 31), suggesting that, ideally, analysts should not only be trained in informatics but in biomedicine also.

Studies in the field of translational research usually collect an abundance of data types: clinical data (demographics, death/survival data, questionnaires, etc.), imaging data (MR, UltraSound, PET, CT, and derived values), biosample data (values from blood, urine, etc.), molecular data (genomics, proteomics, etc.), digital pathology data, data from wearables (blood pressure, heart rate, insulin level, etc.) and much more. To combine and integrate these data types, the scientist needs to understand both informatics (data science, data management, and data curation) and the specific disease area. As there are very many disease areas which all require their own expertise, we will focus here on the informatics side: data integration in translational research. Although this field is relatively new, there are a number of online and offline trainings available. As for the online trainings, Coursera offers a course on “Big Data Integration and Processing” (75). The i2b2 tranSMART Foundation, which is developing an open-source/open-data community around the i2b2, tranSMART and OpenBEL translational research platforms, has an extensive training program available as well (76). As for the offline trainings, ELIXIR offers a number of trainings around data management (77). The European Bioinformatics Institute (EBI) has created a 4-day course specific for multiomics data integration (78).

## Reward and Assessment of Translational Work

In order for the long, collaborative process of discovery toward precision medicine to succeed, it is essential that all involved receive proper recognition and reward. Increasing collaboration means increasing length of authorship which, in turn, highlights the increasing challenges inherent in conventional rewards for intellectual contribution to a publication: in plain terms, if there are over 5,000 authors (79), do only first and last really count? The problem is particularly acute for those working in bioinformatics (80). Encouraging early-career analysts to pursue a biomedical career is challenging if the best they can hope to receive is a mid-author position in a large study. The backdrop to this problem is that similar analyst shortages in other industries have resulted in more alternative options, often better compensated than those in biomedicine (80). Reversing this trend will require substantial changes to biomedical training, with greater emphasis on analysis along with a revised approach to incentives from academic institutions. Trainings such as these in the analysis of big data would enable physicians and researchers to not only enter the Big Data Cycle (Figure 1) on the hypothesis-driven side, but also on the hypothesis-generating side. There is an increasing recognition that traditional methods of assessment and reward are outdated, with an international expert panel convening in 2017 to define six guiding principles toward identifying appropriate incentives and rewards for life and clinical researchers [Table 2 (81)]. While these principles represent a laudable goal, it remains to be seen if and how they might be realized. At some institutions, computational biologists are now promoted for contributing to team scientist as middle authors while producing original work around developing novel approaches to data analysis. Therefore, we would propose adding a seventh principle here: “Developing novel approaches to data analysis.”

**Table 2:** Key proposed principles when assessing scientists.

Addressing societal needs is an important goal of scholarship. Assessing faculty should be based on responsible indicators that reflect fully the contribution to the scientific enterprise. Publishing all research completely and transparently, regardless of the results, should be rewarded. The culture of Open Research needs to be rewarded It is important to fund research that can provide an evidence base to inform optimal ways to assess science and faculty. Funding out-of-the-box ideas needs to be valued in promotion and tenure decisions.

## Summary and Conclusions

In recent years the field of biomedical research has seen an explosion in the volume, velocity and variety of information available, something that has collectively become known as “Big data.” This hypothesis-generating approach to science is arguably best considered, not as a simple expansion of what has always been done, but rather a complementary means of identifying and inferring meaning from patterns in data. An increasing range of “machine learning” methods allow these patterns or trends to be directly learned from the data itself, rather than pre-specified by researchers relying on prior knowledge. Together, these advances are cause for great optimism. By definition, they are less reliant on prior knowledge and hence can facilitate advances in our understanding of biological mechanism through a reductionist “systems medicine” approach. They can also identify patterns in biomedical data that can inform development of clinical biomarkers or indicate unsuspected treatment targets, expediting a goal of precision medicine.

However, in order to fully realize the potential inherent in the Big data we can now generate, we must alter the way we work. Forming collaborative networks—sharing samples, data, and methods—is now more important than ever and increasingly requires building bridges to less traditional collaborating specialities such as engineering, computer science and to industry. Such increased interaction is unavoidable if we are to ensure that mechanistic inferences drawn from Big data are robust and reproducible. Infrastructure capacity will require constant updating, while regulation and stewardship must reassure the patients from whom it is sourced that their information is handled responsibly. Importantly, this must be achieved without introducing stringency measures that threaten the access that is necessary for progress to flourish. Finally, it is clear that the rapid growth in information is going to continue: Big data is going to keep getting Bigger and the way we teach biomedical science must adapt too. Encouragingly, there is clear evidence that each of these challenges can be and is being met in at least some areas. Making the most of Big data will be no mean feat, but the potential benefits are Bigger still.

## Author Contributions

All authors wrote sections of the manuscript. TH and EM put the sections together and finalized the manuscript. DH edited the manuscript.

### Conflict of Interest Statement

TH is employed by Philips Research. SJ is a co-founder of Global Gene Corp, a genomic data platform company dealing with big data. OV acknowledges the financial support of the János Bolyai Research Fellowship, Hungarian Academy of Sciences (Bolyai + ÚNKP-18-4-DE-71) and the TÉT_16-1-2016-0093. RS is currently an employee of Synthetic Genomics Inc., this work only reflects his personal opinions, and he declares no competing financial interest. DH receives research funding or consulting fees from Bristol Myers Squibb, Genentech, Sanofi-Genzyme, and EMD Serono. EM is co-founder and CSO of PredictImmune Ltd., and receives consulting fees from Kymab Ltd. The remaining authors declare that the research was conducted in the absence of any commercial or financial relationships that could be construed as a potential conflict of interest.
